# Perceived stigmatization in cancer patients during the first year after diagnosis: the role of socioeconomic status

**DOI:** 10.1007/s11764-025-01834-0

**Published:** 2025-05-26

**Authors:** Jochen Ernst, Anne-Kathrin Köditz, Ute Goerling, Tanja Zimmermann, Beate Hornemann, Franziska Springer, Anja Mehnert-Theuerkauf

**Affiliations:** 1 Department of Medical Psychology and Medical Sociology, Comprehensive Cancer Center Central Germany (CCCG), University Medical Center Leipzig, Philipp-Rosenthal-Str. 55, D – 04103 Leipzig, Germany; 2 Charité - Universitätsmedizin Berlin, Corporate Member of Freie Universität Berlin, Humboldt-Universität Zu Berlin, and Berlin Institute of Health, Charité Comprehensive Cancer Center, Berlin, Germany; 3 Department of Psychosomatic Medicine and Psychotherapy, MHH — Medizinische Hochschule, Hannover, Germany; 4 Psycho-Oncology, NCT/UCC — National Center for Tumor Diseases Dresden, Dresden, Germany

**Keywords:** Stigmatization, Cancer, Psycho-oncology, Socioeconomic status, Distress

## Abstract

**Purpose:**

Stigmatization in cancer patients leads to poorer health-related outcomes. The effect of socioeconomic status (SES) on stigmatization has not been investigated. We therefore aim to investigate, which differences in stigmatization in cancer patients regarding SES exist up to one year after diagnosis and which medical and demographic characteristics are associated with stigmatization.

**Methods:**

Patients with different solid cancer diagnoses were assessed within two months after diagnosis (*t1*) and at a 12-month follow-up (*t2*). Stigmatization was assessed using the Social Impact Scale (SIS), which comprises four dimensions: *isolation*, *rejection*, s*hame* and *financial insecurity*. An SIS-total score can be computed. Bivariate and multiple regression analysis were performed.

**Results:**

Six hundred-eighty patients completed the assessments (mean age 60.4; 51.2% male; most prevalent cancers: prostate (21.0%), skin (17.1%), breast (16.2%)). Stigmatization at *t1* was moderate in all dimensions, independent of SES. It decreased at *t2*, especially in patients with middle or high SES (*p* < 0.001). Low SES was identified as a risk factor for stigmatization at *t2* (*p* = 0.036 – *p* < 0.001). Other predictors include stigmatization at *t1* as well as distress, disease stage and cancer diagnosis, e.g. lung cancer. The predictors explained 27–43% of the variance in stigmatization in the four dimensions resp. in the SIS-total score.

**Conclusion:**

Stigmatization after cancer diagnosis was dependent of SES. Reasons may be fewer social and economic resources, lower health literacy and insufficient consideration of the specific needs of this patient group in the healthcare system.

**Implication for Cancer Survivors:**

Patients with low SES should receive greater attention in the medical care system and in scientific research to identify and mitigate possible burdens and subsequent problems.

## Introduction

Health-related stigmatization is defined as the attribution of negative characteristics to people because of their illness, typically characterized by social disqualification and devaluation by others [1, 2]. Stigmatization is a multidimensional construct and usually encompasses the four dimensions *social rejection*, *internalized shame*, *financial insecurity* and *social isolation* [3]. There is a distinction between perceived stigmatization and internalized (self-) stigmatization. The latter results from the adoption of a negative external image and can be considered a consequence of perceived stigmatization. Studies that examined external or internalized stigmatization report a stigmatization of cancer patients ranging from 5 to 90% [4, 5]. Beliefs regarding personal responsibility and the controllability of health-related behaviors [6, 7], as well as external visibility of the disease, such as severe disfigurement and changes in body image, can increase stigmatization [8, 9]. Many studies have demonstrated the negative consequences: Stigmatization can delay the utilization of health care services [10, 11] and affect patients’ adherence to treatment, resulting in poorer disease outcomes [2, 12]. A negative impact on quality of life or psychosocial distress has also been identified [13–16]. A previous research has shown that higher levels of stigmatization are associated with sociodemographic and medical factors, such as being female or younger, depression, lower social support, chemotherapy, cancer diagnosis and more advanced stages of cancer, while (full-time) employment, older age, higher income and education tend to mitigate stigmatization [4, 5, 17–20]. The influence of socioeconomic status (SES) — commonly measured using income, education and occupation — as an index variable on cancer-related stigmatization has not been addressed in studies, even though the SES is one of the key determinants of health [21]. So far, only the influence of education and income has been analyzed. Some studies, for example on lung or prostate cancer patients, found no correlation between education, income and stigmatization [16, 22]. Other studies show correlations with education [23] or income [24] or both factors, as determined by the review by Tang et al. [4]. Socioeconomically disadvantaged groups are more likely to develop cancer and to be younger at diagnosis, have a shorter life expectancy after diagnosis and benefit less from treatment [25].

Therefore, it can be assumed that people from lower SES classes are also exposed to greater stigmatization and thus cumulative stress due to their health and social conditions. Yet, prospective data on the development of stigmatization over the course of the disease are scarce and available only for specific diagnostic groups [26].

We therefore analyzed longitudinal data to investigate the following research questions:

1. Which differences in stigmatization in cancer patients regarding SES can be observed within two months after diagnosis *(t1)* and at 12-month follow-up *(t2)*?
2. Which medical and demographic characteristics are associated with stigmatization and can be identified as predictors of stigmatization?

## Materials and methods

### Data collection

Data was collected as a part of the prospective multi-center study “Prevalence of mental disorders, psychosocial distress and need for psychosocial support in cancer patients and their relatives stratified by biopsychosocial factors” with four measurement time points. The study was conducted in the university hospitals (university cancer centers) of the three main German study centers in Leipzig, Berlin and Hannover as well as at cooperating cancer centers in Braunschweig, Dresden, and Göttingen. Study enrolment began in April 2020 and ended in July 2022, follow-up assessments concluded in January 2024. According to the protocol, patients with a solid malignant tumor were included in the study [27] (for diagnosis groups, see Table 1).

**Table 1:** Sample characteristics (N = 680)

Variable			
**Age**	Mean (SD; Range)	60.4 (13.01; 22–90)	
		N*	**(%)**
**Gender**	Female	332	(48.8)
	Male	348	(51.2)
**Socioeconomic status**	High	280	(41.4)
	Middle	296	(43.7)
	Low	101	(14.9)
**Partnership**	Yes	513	(78.9)
	No	137	(21.1)
**Employment status**	Employed (full- or part-time**)**	327	(50.5)
	Retired	293	(45.2)
	Unemployed	13	(2.0)
	Other	15	(2.3)
**Religion**	Yes	186	(28.5)
	No	466	(71.5)
**Residential area**	Urban (≥ 20,000 inhabitants)	378	(57.9)
	Rural (< 20,000 inhabitants)	275	(42.1)
**Diagnostic group (ICD-10)**	Prostate (C61)	143	(21.0)
	Skin (C43, C44)	116	(17.1)
	Breast (C50)	110	(16.2)
	Gynecological (C51-C57)	90	(13.2)
	Gastrointestinal (C15-C25)	81	(11.9)
	Head and neck (C00-C07, C09, C10, C13, C14, C30-C32, C44)	52	(7.6)
	Urinary tract (C64-C68)	43	(6.3)
	Lung (C34)	15	(2.2)
	Other (C41, C45, C48, C49, C60, C62, C71, C78-C80, C97)	30	(4.4)
**Cancer stage (UICC)**	I/II	480	(74.0)
	III/IV	169	(26.0)
**Time since diagnosis**	≤ 1 month	378	(55.6)
	> 1 month	302	(44.4)
**Therapy** **at *t1***	Surgery	599	(89.5)
	Radiotherapy	130	(19.4)
	Chemotherapy	130	(19.4)
	Other	90	(13.5)
	No therapy	11	(1.6)
**Therapy until *t2***	Surgery	317	(46.6)
	Radiotherapy	172	(25.3)
	Chemotherapy	174	(25.6)
	Other	170	(25.0)
	No therapy	114	(16.8)

^*^ Differences to N = 680: missing values

We report the first time point within two months after diagnosis *t1* and the 12-month follow-up *t2*. Given the multiple research questions of the wider project, the issue of stigmatization was only included at those two measurement points. Eligible for study participation were adult patients (≥ 18 years) who were scheduled for cancer treatment at one of the participating cancer centers. Further inclusion criteria were sufficient knowledge of the German language, a physical, psychological and cognitive ability for study participation, and written informed consent as the basis for study participation. Once they had provided informed consent, all participants received a set of validated questionnaires at *t1* and *t2* and were asked to complete the study questionnaires within 10 days. Alternatively, patients had the option of completing the study questionnaires online via an individualized link using the LimeSurvey software [28]. Reminders were sent after two and four weeks to increase the response rate [27]. Exclusion criteria included insufficient German language skills as well as cognitive barriers (e.g., dementia).

### Measures

#### Sociodemographic and medical data

Sociodemographic characteristics such as SES, age, gender, religion, partnership status, residential area and employment status were collected via self-report during an interview with trained research assistants to create data sets that were as complete as possible, given that patients do not always provide valid sociodemographic data (e.g., date of birth, income) in a self-completion questionnaire. Medical data, such as diagnostic group, cancer stage, time since diagnosis and cancer treatment, were obtained from the patients’ medical records (at *t1*) and were recorded using patient questionnaires (at *t2*).

#### Socioeconomic status (SES)

The SES was determined according to Winkler et al. [29], taking into account the characteristics income, education and occupation. Values between 1 and 7 are assigned depending on the individual characteristics. For example, individuals without vocational training are allocated a value of 1 for education, and individuals with a household income of ≥ 4000 € are assigned a value of 7 for income. If a value is missing, it can be substituted by the mean of the values for the other variables. The index can take values from 3 up to 21 and is divided into high (15–21 points), middle (9–14 points) and low (3–8 points) SES.

#### Stigmatization (SIS — Social Impact Scale)

Stigmatization was evaluated using the validated German version of the Social Impact Scale (SIS) [30]. The SIS comprises 24 cancer-related items (response options from 1 “strongly disagree” to 4 “strongly agree”), which are summarized into the following four dimensions (mean score): *Isolation* (Cronbach’s α = 0.92; 9 items), *social rejection* (Cronbach’s α = 0.90; 6 items), *internalized shame* (Cronbach’s α = 0.85; 6 items) and *financial insecurity* (Cronbach’s α = 0.87; 3 items). Items include, for example, “I have the feeling that I am treated with less respect by others than before I had cancer” or “I feel lonely more often now than before I had cancer”. An overall mean (1 thru 4) score can be computed (Cronbach’s α = 0.96). Higher values indicate higher stigmatization and can be divided into “low” (1–1.99), “moderate” (2–2.99) and “severe” (3–4) stigmatization [31, 32].

#### Distress-Thermometer

The DT is a screening tool to assess psychosocial distress in oncology patients using a self-report visual analog scale (range: 0 = no distress, 10 = extreme distress). A cut-off value of ≥ 5 is established, which indicates above-threshold distress and a need for support [33].

### Statistical analyses

We conducted a paired sample *t*-test for the differences in stigmatization at *t1* and *t2* within the SES groups. Analyzes for the SES between-group differences at *t1* and *t2* were performed using a mixed ANOVA and post hoc tests (Duncan). Bivariate analyses of the association between stigmatization and other medical and sociodemographic characteristics were conducted using descriptive methods (frequencies, means, error bars based on standard deviation).

To identify medical and sociodemographic predictors for stigmatization at *t2*, significant predictors from the bivariate analyses were entered in multiple stepwise forward regression models. This was done separately for the stigmatization overall score and for the different dimensions. The analyses were weighted according to SES in order to compensate for the larger proportion of non-participants with low SES and to obtain a distribution of SES analogous to the normal population. Multicollinearity of the included predictors was tested with the indices variance inflation factor (VIF). The critical threshold value for multicolliniarity is specified as ≥ 10 [34]. In our analyses, this value is < 2, indicating that there is no multicollinearity.

The effect size for the *t*-test was reported according to Cohen: 0.20 indicates a weak effect, 0.50 a medium effect and 0.80 a strong effect [35]. The effect sizes for the ANOVA were reported as η^2^ (eta squared) with, according to Cohen, 0.01 indicating a small effect, 0.06 a medium effect, and 0.14 a large effect. Cohen also applies to the explained variance of the regression models: *R*^2^ = 0.02 corresponds to a low variance explanation, *R*^2^ = 0.13 to a moderate and *R*^2^ = 0.26 to a strong variance explanation. Significance tests were two-tailed with an alpha level set at 5%. The analyses were conducted using the IBM SPSS statistical software package (version 29).

## Results

### Sample characteristics

**Fig. 1:**
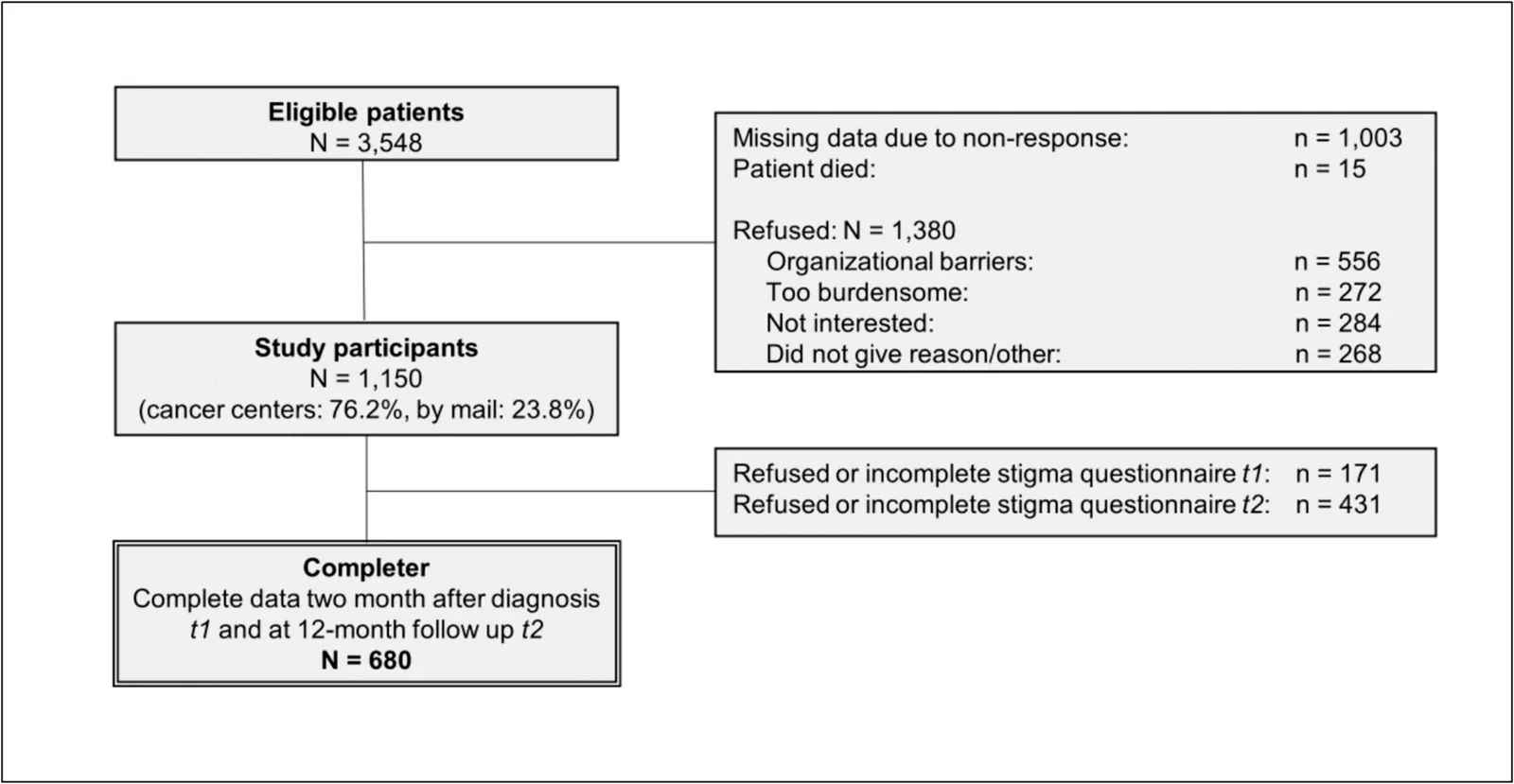
Flowchart of the sample

A total of N = 3,548 eligible patients were contacted, of which *n* = 15 had died before participating in the study and *n* = 2,383 refused to participate or did not respond to the invitation to the study. Reasons for refusal included organizational barriers, burden of the patients or lack of interest. Eventually, *n* = 1,150 were included in the study (Fig. 1). N = 680 completed the questionnaire at *t1* and *t2* and are included in our analysis. The remaining participants did not have complete data sets on stigmatization, refused further participation (*t2*), or did not respond to the invitation to the follow up. Patients had a mean age of 60.4 years, 51.2% were male, 78.9% lived in a partnership. The most prevalent cancers were prostate (21.0%), skin (17.1%) and breast (16.2%) cancer. 74.0% had an UICC stage I-II (Table 1).

#### Stigmatization at t1 and t2 and relationship to SES

Stigmatization at *t1* on all subscales and the total score did not differ between the SES groups and can be rated as “low” with values < 2. Within the SES groups with middle and high SES, stigmatization significantly decreased over time across all dimensions and for the total score (*p* < 0.001), with large effect sizes (d ≥ 0.51). Patients with low SES only showed a decrease over time in two dimensions (*social rejection* and *internalized shame*) and the total score, with effect sizes between d = 0.41 – d = 0.48. For the low SES group, stigmatization remained unchanged for *financial insecurity* and *social isolation* (*p* = 0.914 resp. *p* = 0.435). The between- SES group comparisons show a significantly stronger decrease in stigmatization over time (*t1* to *t2*) in patients with middle and high SES compared to patients with low SES in *social isolation* (*p* < 0.001), *financial insecurity* (*p* < 0.001) and the total score (*p* = 0.002) (see Table 2).

**Table 2:** Changes in stigma over time (*t1, t2*) by SES (Stigma-total score and dimensions; weighted by SES)

	SES: within group comparison*					SES: between group comparison**		
Stigma (SIS) dimensions	*t1*	*t2*	T	|d|	*p*	Measurement time point	F	*p*
**Stigma total score**								
All participants	1.61	1.43	9.18	.51	** <.001**			
Low SES	1.61	1.54	2.01	.41	**.047**	SES—*t1*	.01	.994
Middle SES	1.62	1.43	7.45	.51	** <.001**	SES—*t2*	6.22	**.002**^a)^
High SES	1.61	1.36	5.34	.55	** <.001**			
**Social rejection**								
All participants	1.44	1.26	8.86	.54	** <.001**			
Low SES	1.41	1.29	2.78	.48	**.006**	SES—*t1*	.34	.716
Middle SES	1.45	1.26	7.12	.54	** <.001**	SES—*t2*	1.28	.280
High SES	1.44	1.22	4.62	.56	** <.001**			
**Internalized shame**								
All participants	1.67	1.45	10.70	.56	** <.001**			
Low SES	1.64	1.51	3.05	.47	**.003**	SES—*t1*	.38	.688
Middle SES	1.68	1.43	8.59	.57	** <.001**	SES—*t2*	1.85	.158
High SES	1.70	1.41	5.80	.58	** <.001**			
**Financial insecurity**								
All participants	1.54	1.41	4.93	.68	** <.001**			
Low SES	1.59	1.60	-.11	.72	.914	SES—*t1*	1.94	.145
Middle SES	1.56	1.40	4.64	.68	** <.001**	SES—*t2*	9.36	** <.001**^a)^
High SES	1.44	1.26	3.38	.60	** <.001**			
**Social isolation**								
All participants	1.71	1.55	7.00	.59	** <.001**			
Low SES	1.74	1.70	.78	.49	.435	SES—*t1*	.16	.857
Middle SES	1.70	1.54	5.52	.60	** <.001**	SES—*t2*	7.38	** <.001**^a)^
High SES	1.72	1.45	4.93	.63	** <.001**			

^*^ *t*-Test, ^**^ mixed ANOVA (Post-Hoc-Test: Duncan)

^a)^ Significant for low SES vs. middle and high SES

#### Socio-demographic and medical characteristics associated with stigmatization and predictors of stigmatization

Patients who were younger, unemployed, undergoing chemotherapy or other treatment (*p* < 0.001), and experiencing distress (*t1*: *p* < 0.05, *t2*: *p* < 0.001) were more likely to feel stigmatized at *t1* and *t2* (Table 3). Only at *t1* did higher stigmatization occur in patients diagnosed more than one month previously (*p* < 0.001). Exclusively at *t2,* stigmatization differed by gender (higher for women, *p* < 0.01), by SES (higher for lower SES, *p* < 0.001), and by stage of cancer (higher for advanced stages, *p* < 0.001). The effect sizes are mostly in the small range (η^2^ < 0.06). There was no impact of partnership, religion or place of residence. In general, stigmatization decreased over time. An increase in stigmatization between *t1* and *t2* is only evident in those cases where no oncological treatment had taken place (η^2^ = 0.02).

**Table 3:** **S**tigmatization (SIS-total score) within two months after diagnosis *t1* and at 12-month follow-up *t2* by sociodemographic and medical characteristics (weighted by SES)

Sociodemographic		Stigma total score (mean, sd) *t1*		Stigma total score (mean, sd) *t2*		Δt2/t1*
Age (years)	up to 40	1.82	(.5922)	1.65	(.5260)	-.16
	41–60	1.72	(.6072)	1.50	(.4657)	-.22
	> 60–70	1.54	(.5294)	1.33	(.3397)	-.22
	> 70	1.43	(.4558)	1.31	(.3572)	-.13
		**p <.001, η**^**2**^** =.05**		**p <.001, η**^**2**^** =.07**		
**Gender**	Male	1.61	(.5808)	1.36	(.3941)	-.24
	Female	1.61	(.5489)	1.47	(.4521)	-.14
		n.s., η^2^ =.00		**p <.01, η**^**2**^** =.02**		
**Socio-economic status**	Low	1.61	(.5148)	1.54	(.4891)	-.07
	Middle	1.62	(.5935)	1.43	(.4269)	-.19
	High	1.61	(.5545)	1.36	(.3920	-.25
		n.s., η^2^ =.00		**p <.001, η**^**2**^** =.02**		
**Partnership**	Yes	1.62	(.5719)	1.41	(.4250)	-.22
	No	1.59	(.5463)	1.45	(.4209)	-.14
		n.s., η^2^ =.00		n.s., η^2^ =.00		
**Employment status**	Employed (full- or part-time)	1.70	(.5866)	1.48	(.4588)	-.22
	Retired	1.51	(.5233)	1.33	(.3637)	-.18
	Unemployed	1.97	(.4713)	1.59	(.4350)	-.38
	Other	1.59	(.5282)	1.46	(.3970)	-.13
		**p <.001, η**^**2**^** =.04**		**p <.001, η**^**2**^** =.03**		
**Religion**	Yes	1.55	(.5482)	1.46	(.4428)	-.10
	No	1.64	(.5711)	1.40	(.4149)	-.24
		n.s., η^2^ =.01		n.s., η^2^ =.00		
**Residential area**	Urban (≥ 20,000 inhabitants)	1.62	(.5640)	1.41	(.3954)	-.21
	Rural (< 20,000 inhabitants)	1.61	(.5688)	1.42	(.4619)	-.18
		n.s., η^2^ =.00		n.s., η^2^ =.00		
**Medical**						
Therapy**	Surgery	1.62	(.5648)	1.40	(.4174)	-.22
	Radiotherapy	1.65	(.6238)	1.45	(.4338)	-.21
	Chemotherapy	1.76	(.5903)	1.55	(.4184)	-.21
	Other	1.79	(.6396)	1.46	(.4133)	-.34
	No therapy	1.31	(.4071)	1.59	(.6222)	.28
		***p* <.001, η**^**2**^** =.00-.02**		***p* <.001, η**^**2**^** =.00-.03**		
**Cancer stage (UICC)**	I/II	1.61	(.5734)	1.36	(.3936)	-.25
	III/IV	1.68	(.5507)	1.61	(.4605)	-.07
		n.s., η^2^ =.00		***p* <.001, η**^**2**^** =.07**		
**Time since diagnosis**	up to 1 month	1.51	(.5340)	1.40	(.4373)	-.11
	> 1 month	1.74	(.5787)	1.44	(.4120)	-.30
		***p* <.001, η**^**2**^** =.04**		n.s., η^2^ =.00		
**Distress**	Distress *t1* and *t2*	1.78	(.5573)	1.77	(.5065)	-.01
	Distress *t1*	1.66	(.5465)	1.46	(.3851	-.20
	Distress *t2*	1.60	(.5627)	1.46	(.4167)	-.14
	No distress *t1* and *t2*	1.55	(.5662)	1.27	(.3275)	-.29
		***p* <.01, η**^**2**^** =.02**		***p* <.001, η**^**2**^** =.18**		

^*^ Negative value: decrease in stigmatization from *t1* to *t2*

^**^ Multiple responses possible

**Fig. 2:**
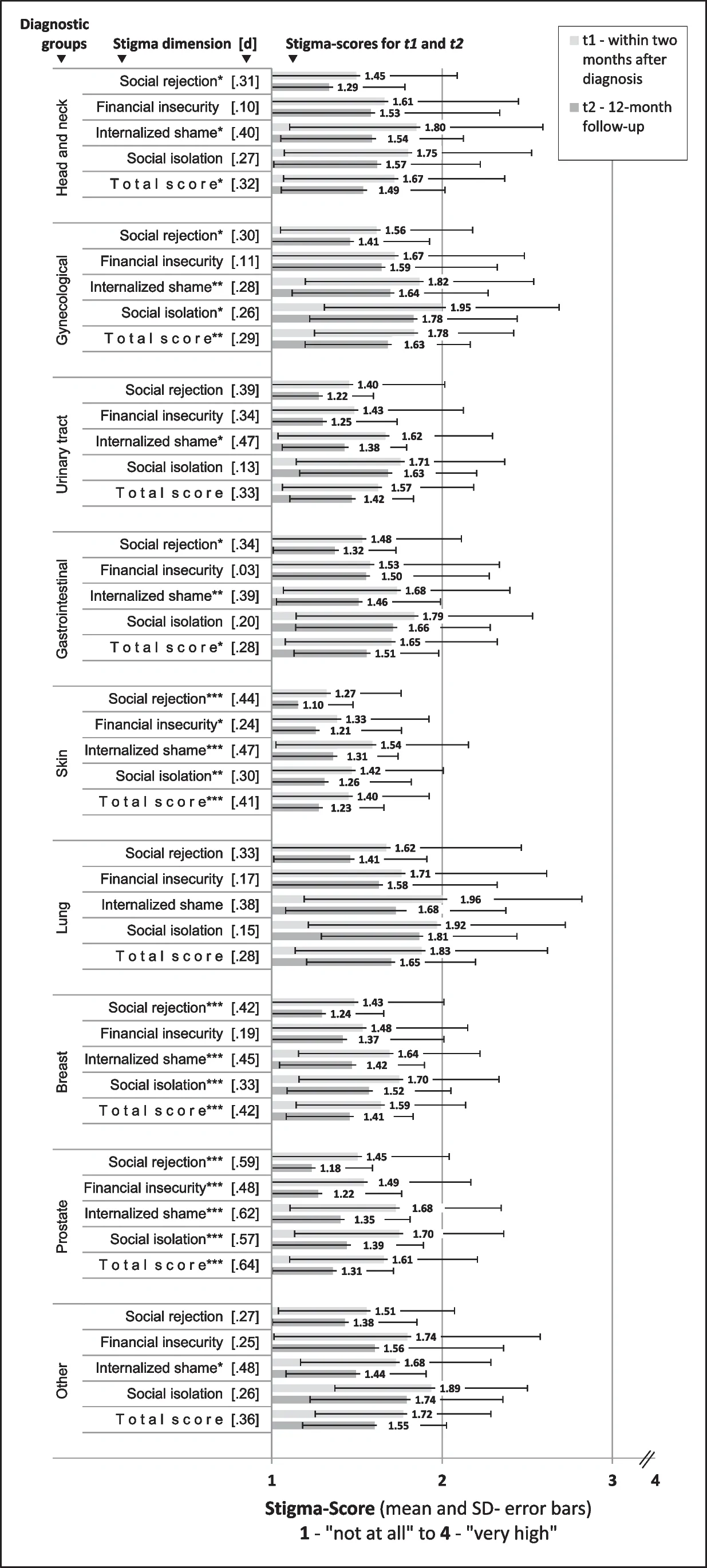
Stigmatization within two months after diagnosis *t1* and at 12-month follow-up *t2* (dimensions and SIS-total score) by diagnostic groups. Change in significance *t2*—*t1*: * = p < 0.05 ** = *p* < 0.01 *** = *p* < 0.001; in []: effect size Cohen’s |d|

The level of stigmatization differed significantly between diagnostic groups (Fig. 2). The cancers with the highest stigmatization were lung cancer (SIS-total score: mean *t1* = 1.83; mean *t2* = 1.65) and gynecological tumors (SIS-total score mean *t1* = 1.78; mean *t2* = 1.63), whereas patients with skin (SIS-total score mean *t1* = 1.40; mean *t2* = 1.23) and prostate cancer (SIS-total score mean *t1* = 1.61; mean *t2* = 1.31) showed the lowest stigmatization. Respondents felt most stigmatized in the dimension of *internalized shame* at *t1* and *social isolation* at *t2*. Stigmatization decreased over time across all dimensions and diagnostic groups with varying degrees. There was a significant reduction in the dimension *internalized shame* in 8/9 diagnostic groups (*p* < 0.05 to *p* < 0.001) and *social rejection* in 6/9 diagnostic groups (*p* < 0.05 to *p* < 0.001), as well as in the SIS-total score in 6/9 diagnostic groups (*p* < 0.05 to *p* < 0.001). The effect sizes for the diagnosis groups are for the most part in the range of d > 0.02. The effect sizes are greatest for prostate cancer (almost all with a medium size d > 0.05), and this patient group also shows the greatest reduction in stigmatization at follow-up.

The results of the regression models (dependent variable: stigmatization at *t2*) differentiated according to the SIS-total score, and the four subscales of the SIS are shown in Table 4. Patients with middle or high SES perceived significantly less overall stigmatization (*p* = 0.036 and *p* < 0.001 respectively), less *financial insecurity* (*p* = 0.006 and *p* < 0.001 respectively) and less *social isolation* (*p* = 0.007 and *p* < 0.001 respectively) at *t2* than patients with low SES. The predictors “stigmatization at *t1*” and “distress at *t1 and t2*” had a positive effect on stigmatization in all dimensions and the SIS-total score (*p* < 0.001). Negatively associated with stigmatization was “retired” (*financial insecurity*, *p* < 0.001). Medical factors partly increased stigmatization: for most of the dimensions, this was linked with a more advanced cancer stage (*p* ≤ 0.001). Gastrointestinal cancer was associated with higher stigmatization in the dimension of *financial insecurity* (*p* < 0.019), gynecological cancer in two dimensions (*social rejection and internalized shame*, *p* = 0.011 and *p* = 0.006 respectively). “Other therapy *t1*” reduced the stigmatization in the dimension of *internalized shame*, whereas a diagnosis of lung cancer increased it (*p* = 0.018 and *p* = 0.036 respectively). “Chemotherapy *t2*” led to higher levels of stigmatization in overall stigmatization and in the *social isolation* dimension (*p* = 0.018 and *p* = 0.031 respectively), whereas “no therapy *t2*” had a mitigating effect (*p* = 0.037). A diagnosis of skin cancer lowered stigmatization in two dimensions (SIS-total score and *social isolation*: *p* = 0.017 and *p* = 0.003 respectively). The explained variance *R*^2^ was high, ranging from 0.27 to 0.43.

**Table 4:** Multivariate analysis (Regression): Stigmatization within two months after diagnosis *t1* and 12-month follow-up *t2* (SIS-total score) by sociodemographic and medical predictors

*Predictors*		*in []: Reference*	**B**	***ß***	***p***	**95% CI**	
**Stigma total score** (*adj. Nagelkerkes R*^2^ = *.43)*							
Middle SES		[low SES]	-.073	-.083	**.036**	-.140	-.005
High SES		[low SES]	-.135	-.129	**.001**	-.217	-.053
Stigma *t1 *(total score)			.309	.415	** <.001**	.262	.356
Distress *t1* and *t2*		[no]	.387	.342	** <.001**	.317	.457
Cancer stage III or IV		[I or II]	.145	.150	** <.001**	.079	.210
Chemotherapy *t2*		[no]	.077	.082	**.018**	.013	.140
Surgery *t2*		[no]	.067	.078	**.015**	.013	.121
Skin cancer		[no]	-.098	-.080	**.017**	-.179	-.018
**Social rejection** (*adj. Nagelkerkes R*^2^ = *.27)*							
Stigma *t1* (total score)			.239	.345	** <.001**	.190	.239
Distress *t1* and *t2*		[no]	.256	.243	** <.001**	.182	.256
Cancer stage III or IV		[I or II]	.159	.177	** <.001**	.096	.159
Gynecological cancer		[no]	.098	.091	**.011**	.023	.098
**Financial insecurity** (*adj. Nagelkerkes R*^2^ = *.27)*							
Middle SES	[low SES]		-.168	-.125	**.006**	-.287	-.049
High SES	[low SES]		-.304	-.187	** <.001**	-.448	-.160
Retired	[no]		-.304	-.230	** <.001**	-.399	-.210
Stigma *t1*(total score)			.330	.286	** <.001**	.248	.412
Distress *t1* and *t2*	[no]		.310	.177	** <.001**	.186	.433
Cancer stage III or IV	[I or II]		.175	.117	**.001**	.069	.280
No therapy *t2*	[no]		-.169	-.093	**.009**	-.295	-.042
Gastrointestinal cancer	[no]		.164	.083	**.019**	.027	.301
**Internalized shame** (*adj. Nagelkerkes R*^2^ = *.30****)***							
Stigma *t1* (total score)			.339	.421	** <.001**	.283	.395
Distress *t1* and *t2*	[no]		.331	.270	** <.001**	.247	.414
Other therapy *t1*	[no]		-.106	-.083	**.018**	-.194	-.018
Gynecological cancer	[male]		.122	.097	**.006**	.036	.208
Lung cancer	[no]		.207	.073	**.036**	.014	.401
**Social isolation** (*adj. Nagelkerkes R*^2^ = *.40)*							
Middle SES	[low SES]		-.125	-.111	**.007**	-.216	-.034
High SES	[low SES]		-.200	-.147	** <.001**	-.310	-.090
Stigma *t1* (total score)			.320	.331	** <.001**	.257	.382
Distress *t1* and *t2*	[no]		.521	.354	** <.001**	.427	.614
Cancer stage III or IV	[I or II]		.204	.163	** <.001**	.118	.290
Chemotherapy *t2*	[no]		.096	.079	**.031**	.009	.184
No therapy *t2*	[no]		-.137	-.090	**.008**	-.238	-.035
Radiotherapy *t2*	[no]		-.103	-.075	**.025**	-.193	-.013
Skin cancer	[no]		-.166	-.103	**.003**	-.274	-.057

## Discussion

This study investigated the stigmatization and its relation to SES in N = 680 cancer patients with solid tumors from nine different diagnostic groups within two months after diagnosis *(t1)* and at 12-month follow-up *(t2)*. Other medical and sociodemographic characteristics were analyzed as possible predictors.

Analyses show that stigmatization at *t1* (with a possible range of 1–4) for the SIS-total score and the four dimensions is “low”, ranging from 1.44 to 1.71, and does not differ between SES groups. We observed a decrease in all dimensions of stigmatization over time in patients with middle and high SES (*p* < 0.001). Among patients with low SES, however, stigmatization remained unchanged or decreases less than in patients with higher SES. There is only limited comparative data on the course of stigmatization over time. A cohort analysis reports a moderate decrease in stigmatization over time as a result of adapting to the disease and activating resources [5]. Patients with higher SES usually have better health literacy and more social and economic resources for addressing stigmatization, whereas people with low SES are more likely to face disadvantageous living conditions and lower social inclusion [36]. Health literacy often moderates access to and use of medical services [37]. By providing comprehensible information and facilitating the development of relevant skills, e.g. as part of target group-specific interventions to increase health literacy, a contribution can be made to reducing disadvantage and stigmatization among patients with low SES [38]. The latter are less likely to seek professional support and are more likely to have a less supportive social network and greater financial problems and worries [39]. These can also be experienced as stigmatizing [40] and may thus contribute to the persistence of stigmatization. In order to support patients with low SES in reducing stigmatization and its consequences, it is important that healthcare providers communicate empathetically with patients, using appropriate language to minimize potential stigma experiences. The aim should also be to assess the information status as well as the health and digital literacy of these patients and to counteract potential social inequalities [41, 42]. Furthermore, employees of the healthcare system should inform proactively and directly on available benefits, such as statutory health insurance providers covering the costs of certain medical treatments or the elimination of co-payments for medication for low-income patients, in order to reduce financial burdens and further stigmatization among this patient group. In accordance with previous research, in the univariate analyses we found younger age and being unemployed to be stigma-increasing [4, 5, 26, 43]. For younger people, the physical, social or psychological limitations associated with a cancer diagnosis are less socially expected and accepted. Added to this are self-stigmatization, feelings of shame or (as in the case of unemployed people, for example) additional work-related stigmatization. With regard to medical factors, such as distress, therapy, time since diagnosis, diagnostic group or tumor stage, our results are also consistent with previous research. For example, increased distress, chemotherapy, diagnosis within 3 months, lung, gynecological and head and neck cancer are considered characteristics that increase stigmatization. [8–10, 15, 18, 19, 44]. In addition to heightened psychosocial and physical distress and maladaptive coping, the visibility of the cancer (e.g., alopecia, changes in body image [45]) and beliefs regarding personal responsibility and the controllability of health-related behaviors, e.g., in lung cancer, contribute to higher levels of stigmatization [20].

The results that have been discussed so far could be replicated in our multivariate predictor analyses. In addition to SES, it is mainly medical characteristics that influence stigmatization. Patients with low SES showed a higher overall stigmatization as well as in the dimensions of *financial insecurities and social isolation*. The strongest further predictors in all SIS dimensions were stigmatization already at baseline and psychosocial distress. Other studies have also shown a close association between the two constructs, although no causal relationships could be derived from the previous predominantly cross-sectional approaches [46, 47] or distress was assessed as a consequence of stigma, not as a cause [48]. Further, more extensive studies are needed here in order to clarify the link between stigma and distress. Advanced cancer stage and chemotherapy were relevant factors for increased stigmatization in some SIS dimensions, and “no current therapy” reduced the risk of stigmatization. This is largely due to the visibility of the disease or the consequences of treatment, such as chemotherapy [18]. In contrast, patients with skin cancer are less stigmatized. This form of cancer is not visible in many cases and, in addition, bodily functions are less restricted and the prognosis tends to be more favorable [26]. In the dimension of *internalized shame*, patients with lung cancer in particular felt stigmatized. Shame, especially as a result of the feeling that the illness is “self-inflicted”, as shown in the literature [49, 50], may be one reason, as patients who mentioned internal causal attributions reported poorer psychological adjustment und greater stigmatization. This is more often the case in in lung cancer patients compared to breast or prostate cancer patients.

## Limitations

Despite intensive efforts, it has been difficult to achieve balanced SES stratification when recruiting patients, as people with low SES often refuse to participate. Our approach of particularly motivating patients with low SES during recruitment or using adapted, simpler language to attract them to the study and offer support could not be maintained due to the coronavirus pandemic and restricted access to clinics (potential study participants were only allowed to be contacted by post during lockdown). This also applies to the follow-up analysis, as this group does not tend to participate in repeat surveys. Consequently, we probably underestimated the impact of SES in our analyses, insofar as patients with a tendency towards higher stigmatization (low SES) did not participate in the study. It is possible that the multiple study approach (interviews, questionnaires) could have contributed to a higher drop-out rate, insofar as there was more effort involved for the patients. However, we do not consider this bias to be significantly greater than is usually the case with questionnaire surveys. Conducting an interview with personal contact can also contribute to an increase in participation, as patients perceive appreciation and interest in their own problems in the context of the cancer diagnosis. In our study, only patients with solid cancers were surveyed; further specific correlations may apply to hematological diagnostic groups. This concerns, for example, the numerous therapy-associated risks in these diagnostic groups, a high mortality and recurrence rate, invasive companion diagnostics and the need for long-term behavioral changes in various areas of life. We are planning further studies in this area.

There were few patients in some diagnostic groups (e.g., lung cancer), so the results should be interpreted with caution. The majority of respondents (74%) had early stage disease (UICC I-II), as patients with a high disease burden tend to reject participation in studies. This probably led to an underestimation of the impact of disease severity.

To our knowledge, our study is the first survey with a large, homogeneous sample of cancer patients with regard to the time since diagnosis that longitudinally examines the topic of stigmatization with a focus on SES. The process-oriented approach (two measurement points) makes it possible to show developments and changes in stigmatization within one year of the cancer diagnosis and to examine which characteristics are associated with stigmatization.

## Conclusion

Stigmatization was moderate in all dimensions at *t1* (diagnosis), regardless of SES. After one year, a decrease can only be observed in people with higher and medium SES, while stigmatization persists in people with low SES. In addition to distress, other predictors of stigmatization include medical characteristics such as cancer stage, therapy and diagnosis group. In the course of care, it is important to pay particular attention to patients with low SES, as they often have fewer resources to deal with stigmatization. Low-threshold access to healthcare services, appropriate information and education as well as greater social support and guidance in the medical care system promote health literacy and can help to reduce stigmatization and its negative consequences, especially in people with low SES.

## Data Availability

The datasets generated during and/or analysed during the current study are available from the corresponding author on reasonable request.
